# Immune Cell Profiles and Novel Insights Into Cancer Risk: A Focus on Oral and Pharyngeal Cancer

**DOI:** 10.1155/humu/6585175

**Published:** 2026-07-09

**Authors:** Xin Deng, Shaohong Huang

**Affiliations:** ^1^ Stomatological Hospital, School of Stomatology, Southern Medical University, Guangzhou, Guangdong, China, fimmu.com

**Keywords:** causal inference, flow cytometry, genome-wide association studies, immune cells, Mendelian randomization, oral and pharyngeal cancer

## Abstract

**Background and Objective:**

Oral and pharyngeal cancers affect 850,000 new cases annually with 440,000 deaths worldwide. This study explores the causal relationship between immune cell profiles and oral‐pharyngeal cancer risk through Mendelian randomization analysis integrated with experimental validation.

**Methods:**

A two‐sample Mendelian randomization study was conducted using the MRC IEU OpenGWAS platform, analyzing data from 3757 European individuals for immune cell profiles and 4671 oral‐pharyngeal cancer cases, with 80% statistical power to detect OR ≥ 1.2. Multiple Mendelian randomization methods were employed, including inverse‐variance weighted, MR‐Egger, and weighted median approaches. Findings were validated through coculture experiments examining immune cell‐cancer cell interactions via flow cytometry.

**Results:**

Among 731 immune traits analyzed, Mendelian randomization revealed elevated cancer risk associated with CD127 on CD45RA+ CD4+ T cells (OR = 1.33, 95% CI: 1.04–1.70, *p* = 0.025, F − statistic = 53.08), CD4 on CD39+ CD4+ T cells (OR = 1.17, 95% CI: 1.01–1.35, *p* = 0.032, *F* = 34.88), and HLA‐DR on CD8+ T cells (OR = 1.25, 95% CI: 1.04–1.50, *p* = 0.018, *F* = 50.99). Flow cytometry validation (*n* = 3 independent experiments, five replicates each) demonstrated that memory B cells enhanced cancer cell viability (92.95*%* ± 3.2*%* at 72 h, *p* < 0.001) while inhibiting apoptosis. Conversely, naive CD8+ T cells showed antitumor effects, reducing cancer cell viability to 67.99*%* ± 2.8*%* and inducing apoptosis (28.42*%* ± 1.9*%* vs. 12.35*%* ± 1.2*%* control, *p* < 0.001).

**Conclusion:**

This integrated analysis not only reveals novel immune cell‐cancer risk associations and their underlying mechanisms but also provides promising opportunities for personalized immunotherapy development in oral‐pharyngeal cancer. These findings enable precise patient stratification based on immune cell profiles and suggest tailored therapeutic strategies that could significantly improve clinical outcomes.

## 1. Introduction

According to recent global cancer statistics, oral and pharyngeal cancers constitute a significant and growing portion of the global cancer burden, with an estimated 850,000 new cases and 440,000 deaths annually worldwide. Recent studies in oncoImmunology have highlighted the critical role of immune cells in cancer development, and our research is aimed at delving deeper into the specific immune cell profiles associated with oral and pharyngeal cancer, building upon the foundation laid by previous investigations in this field [1–3].

The relationship between immune function and cancer development has emerged as a central focus in tumor immunology research [4–6]. Although previous studies focused on cell proportions, our analysis of surface marker densities and median fluorescence intensities provides quantitative insights into immune cell activation states relevant to tumor surveillance. Specifically, CD127 expression indicates T cell survival capacity critical for antitumor memory, CD39 marks immunosuppressive cells generating adenosine in the tumor microenvironment, and HLA‐DR on CD8+ T cells represents activation and potential exhaustion status. It has been found that chronic inflammation, often mediated by immune cells, can create an environment in the oral cavity and pharynx that is favorable for cancer development [7]. Specifically, certain proinflammatory molecules released by cells have been implicated in promoting the formation of cancer cells [8].

Recent advances in tumor immunology have revealed that myeloid‐derived suppressor cells (MDSCs) and regulatory T cells (Tregs) have contributed to oral and pharyngeal cancers′ rapid progression and poor prognosis [9]. These cell types can suppress our bodies antitumor immune response allowing cancer cells to evade detection by the immune system [10]. Previous studies have shown that alterations in the function of killer cells (NK) and specific B‐cell and T‐cell populations are involved in developing oral and pharyngeal cancers [11]. The complex interplay between complex species of immune cells and cancer cells is crucial because it can either hinder or promote tumor growth [12].

Building upon decades of cancer immunology research from foundational studies [1–3] to recent clinical applications [13–15], our work applies causal inference methods to distinguish correlation from causation in immune‐cancer relationships. In contrast to conventional observational studies that are often confounded by various factors, Mendelian randomization (MR) offers a more robust approach to disentangling the causal relationship between immune cells and cancer risk. Although prior research has identified associations between immune cell activity and oral and pharyngeal cancers, most findings are based on observational studies, which are inherently limited in determining causality. These studies are prone to confounding factors and reverse causation, making it challenging to identify whether immune cell behavior directly contributes to cancer risk. To address these limitations, we employed MR, a method that uses genetic variants as instrumental variables (IVs) to mimic a randomized control trial [13]. By capitalizing on genetic variants as IVs, we can gain a more accurate understanding of the underlying mechanisms. The advantage of using MR is that it provides a detailed understanding of the relevant causal pathways, thereby revealing the intricate link between the immune system and cancer and providing a key guide to cancer prevention and treatment [14, 15].

This method mimics the design of a randomized control trial. In a natural setting, providing a more reliable prediction of the causal effect of risk factors (in this study, specific immune cells) on disease outcomes [16]. By employing this approach our research is aimed at answering the question of whether and how specific immune cells play a causal role in influencing the risk of developing oral and oropharyngeal cancers.

In order to accomplish our research goals, we utilized a technique called two‐sample randomization. This method involves using genetic variants as substitutes for modifiable exposures (specific immune cells) to determine their impact on the risk of developing oral and pharyngeal cancers. We identified various IVs of immune cells based on genome‐wide association studies (GWAS). Our main aim is to understand how immune cells function in cancer pathogenesis, which will contribute to a better understanding of oral and pharyngeal cancer′s origins. This study has the potential to uncover targets for prevention and treatment strategies, ultimately leading to improved clinical outcomes for patients suffering from these debilitating diseases.

## 2. Materials and Methods

### 2.1. Guidelines and Study Design

We examined how various immune cell types impact the occurrence of oral and pharyngeal cancers through a two‐sample MR analysis. The analysis utilized publicly available datasets and adhered to the Strengthening the Reporting of Observational Studies in Epidemiology—Mendelian randomization (STROBE‐MR) guidelines, as illustrated in Figure S1. This study was conducted in accordance with the ethical principles of the Declaration of Helsinki and was approved by the Ethics Committee of the Stomatological Hospital, Southern Medical University. Data protection followed GDPR guidelines with all analyses performed on deidentified summary statistics. All cell lines and primary cells used in this study were obtained from established biological repositories: the oral squamous cell carcinoma cell line CAL‐27 was sourced from the National Infrastructure of Cell Line Resource (Beijing, China, Catalog #3131C0001000700122), and peripheral blood mononuclear cells (PBMCs)were purchased from LONZA (Basel, Switzerland, Catalog #CC‐2702). No additional ethical approval was required for the use of these commercially available cell lines. Clinical trial number was not applicable, as this study utilized commercially available cell lines, antibodies, and public datasets that did not require clinical trial registration.

### 2.2. Sources of Data

Data on immune cells were derived from GWAS involving 3757 European participants, using publicly available datasets from the MRC IEU OpenGWAS platform. This platform was specifically selected for its comprehensive coverage of genetic associations and rigorous quality control measures, including standardized data formatting, harmonization protocols, and extensive documentation of study‐level metadata. The platform′s regular updates and maintenance of genetic association data ensure data reliability and reproducibility. Immune cell traits were identified based on a prior immune‐cell‐related study [17], and 731 traits were selected for analysis (Table S1). Oral and pharyngeal cancer GWAS data (GWAS ID: IEU‐B‐90) were retrieved from the same platform, encompassing 4671 European samples [18]. To account for potential confounding, smoking‐related traits (*n* = 93) were also obtained from GWAS data (Table S2). All standardized association statistics were extracted using the R package TwoSampleMR [19]. The use of data from public databases (MRC IEU OpenGWAS platform) in this study was reviewed and approved by the Ethics Committee of the Stomatological Hospital, Southern Medical University. The committee confirmed that no additional ethical approval was required for the analysis of these publicly available, deidentified datasets.

### 2.3. IVs Selection

This study employs a genetic variant as an IV that satisfies three essential conditions. First, the chosen IV demonstrates a significant correlation with the exposure factor. Second, the IV is not significantly correlated with potential confounders that could impact the exposure or outcome. Third, the IV influences the outcome exclusively through the “IV → Exposure → Outcome.” This establishes the validity of the genetic variant as an IV, ensuring that other variables do not confound the exposure effect estimates on the outcome.

This study initially selected single nucleotide polymorphisms (SNPs) with a *p* value less than 5 × 10^−8^ in the GWAS of the exposure group as potential IVs. SNPs exhibiting linkage disequilibrium (LD) were excluded to refine this selection. This was done by removing SNPs with a physical distance greater than 10,000 kb and an *r* ^2^ less than 0.001. Subsequently, the GWAS data were analyzed to calculate the F‐statistic using the following formula, which assesses the bias due to weak IVs. Any findings with an F‐statistic value less than 10 were excluded to minimize the potential bias in the results caused by weak IVs. This approach ensures the robustness of the IV analysis by mitigating the influence of weak instruments on the estimation of causal effects [20]:

F=N−k−1k×R21−R2

*N* represents the size of the sample, *K* denotes the quantity of IVs employed, and the *R*‐squared value indicates how well the IVs account for the exposure. *R* ^2^ = 2 × (1 − MAF) × MAF ×  ^²^ *β*, where MAF is the minimum allele frequency and *β* is the allele effect size. This formula can be simplified to *F* = *β* ^2^/SE^2^ when directly using the beta coefficient and standard error from GWAS summary statistics, which is mathematically equivalent and more commonly applied in practice.

Using this approach, all individual SNP F‐statistics in our study exceeded the conventional threshold of 10, with values ranging from 30.09 to 2037.04 across all IVs. Table 1 presents the median F‐statistic for each immune marker along with the minimum and maximum values, with median values ranging from 32.78 to 108.09, indicating robust instrument strength for all genetic variants used in our analysis. The detailed SNP‐level data including individual F‐statistics for all IVs are available from the IEU OpenGWAS database (Dataset IDs: GCST90001391‐GCST90002122 for immune traits, ieu‐b‐90 for oral‐pharyngeal cancer) and can be provided as supporting information upon request.

**Table 1:** Immune cell markers analysis results for oral and pharyngeal cancer.

Immune cell marker	Instrumental variable strength		Mendelian randomization analysis				Steiger directionality			Heterogeneity test			Pleiotropy test		
	SNPs	Median F (min–max)	Beta	SE	OR (95% CI)	*p* value	SNP *r* ^2^ exposure	SNP *r* ^2^ outcome	Correct direction	Q	Q_df	Q_pval (*I* ^2^ *%*)	MR‐Egger intercept	SE	*p* value
BAFF‐R on IgD+ CD38dim B cell	7	108.09 (71.01–2037.04)	0.110	0.047	1.12 (1.02–1.22)	0.020	0.382197	0.001844	TRUE	3.175393	6	0.786533 (−88.95)	0.05832	0.055051	0.337895
CD127 on CD45RA+ CD4+ T cell	3	53.08 (47.72–54.24)	0.282	0.126	1.33 (1.04–1.70)	0.025	0.052251	0.001234	TRUE	0.745084	2	0.688981 (−168.43)	0.06467	0.299707	0.864713
CD20 on IgD+ CD38dim B cell	6	41.32 (30.14–54.61)	0.273	0.114	0.76 (0.61–0.95)	0.017	0.068243	0.002299	TRUE	5.041632	5	0.410821 (0.83)	0.03651	0.132229	0.796114
CD4 on CD39+ CD4+ T cell	6	34.88 (30.09–350.54)	0.156	0.073	1.17 (1.01–1.35)	0.032	0.17569	0.001623	TRUE	3.006071	5	0.69905 (−66.33)	0.018715	0.054338	0.747878
HLA DR on CD33+ HLA DR+ CD14dim	2	37.71 (35.34–40.07)	0.542	0.231	0.58 (0.37–0.91)	0.019	0.046783	0.001824	TRUE	1.293211	1	0.255457 (22.67)	—	—	—
HLA DR+ CD8+ T cell %lymphocyte	3	50.99 (35.85–280.72)	0.223	0.094	1.25 (1.04–1.50)	0.018	0.096391	0.001426	TRUE	0.989594	2	0.609695 (−102.10)	0.082556	0.088925	0.523637
HLA DR+ CD8+ T cell absolute count	4	51.56 (30.72–237.56)	0.218	0.090	1.24 (1.04–1.49)	0.016	0.099265	0.001441	TRUE	0.887038	3	0.828555 (−238.20)	0.003126	0.064696	0.965859
HLA DR++ monocyte %monocyte	3	43.11 (39.36–98.83)	0.381	0.115	0.68 (0.55–0.86)	0.001	0.049478	0.0024	TRUE	0.05412	2	0.973303 (−3595.46)	0.003691	0.139792	0.983193
IgD on IgD+ CD38+ B cell	2	48.78 (44.87–52.68)	0.449	0.202	1.57 (1.05–2.33)	0.026	0.02635	0.001105	TRUE	0.202709	1	0.652543 (−393.32)	—	—	—
Memory B cell %B cell	2	32.78 (31.39–34.17)	1.236	0.386	3.44 (1.61–7.33)	0.001	0.017783	0.002346	TRUE	0.685172	1	0.407812 (−45.95)	—	—	—
Naive CD8+ T cell %T cell	2	33.31 (31.07–35.55)	0.746	0.231	0.47 (0.30–0.75)	0.001	0.019272	0.002396	TRUE	0.752394	1	0.385719 (−32.91)	—	—	—

*Note:* The *I* ^2^ statistic reflects the proportion of the heterogeneous part of the instrumental variable in the total variation.

Abbreviations: *r* ^2^, variance explained rate; CI, confidence interval; OR, odds ratio; Q, Cochran′s Q test statistic; Q_df, Q test degree of freedom; Q_pval, *p* value of Q test; SNP, single nucleotide polymorphism.

### 2.4. MR Causal Effect Estimation

The study employed a two‐sample MR approach to explore the causal influence of immune cells on the susceptibility to oral cavity and pharyngeal cancer. The primary method for estimating MR causal effects was the inverse variance weighted (IVW) technique. Additionally, we implemented four alternative approaches: MR‐Egger, weighted median (WME), simple mode (SM), and weighted mode (WM), each relying on distinct identifying assumptions to ensure result robustness. For multiple comparisons, multiple testing was adjusted using the false discovery rate (FDR) with Benjamini–Hochberg procedure, considering *q* − value < 0.1 as significant. Sample size calculations indicated 80% power to detect odds ratio (OR) ≥ 1.2. Missing data (<2% of summary statistics) were handled through complete case analysis after confirming the missing completely at random assumption.

We employed multiple approaches to assess pleiotropy: (1) MR‐Egger intercept test for horizontal pleiotropy, (2) MR‐PRESSO to identify and remove outlier SNPs, (3) multivariable MR adjusting for smoking using pack‐years data from the IEU OpenGWAS database (Table S2), (4) leave‐one‐out analysis for influential variants, and (5) Steiger directionality test to confirm causal direction.

All analyses were performed using R Version 4.3.2 with the following packages: TwoSampleMR (v0.5.7), MR‐PRESSO (v1.0), and MendelianRandomization (v0.9.0). GWAS data were accessed from the IEU OpenGWAS database (build 38, accessed January 2025). Flow cytometry data were analyzed using FlowJo v10.8.1.

### 2.5. Sensitivity Analysis

The study conducted comprehensive sensitivity analyses to ensure the robustness of causal estimates and address potential violations of MR assumptions.

Heterogeneity assessment: Initially, heterogeneity among SNP estimates was assessed using the Cochran Q test. For results with significant heterogeneity, the iIVW random effects model was employed to estimate the magnitude of causal effects. The Cochran Q‐test is limited to assessing the presence of heterogeneity, rather than its distribution. Additionally, the study utilized the *I* ^2^ statistic to evaluate the extent of heterogeneity, where an *I* ^2^ of ≤ 0% indicates no heterogeneity, 0%–25% suggests low heterogeneity, 25%–50% indicates moderate heterogeneity, and >50% signifies high heterogeneity. This methodological approach ensures a nuanced understanding of the variability in the causal estimates and strengthens the reliability of the study′s conclusions.

I2=Q−dfQ×100%

#### 2.5.1. Pleiotropy Assessment

Subsequently, the study employed multiple approaches to assess pleiotropy. First, the MR‐Egger method was used to test for horizontal pleiotropy among the IVs, with the intercept term providing a formal test where. A *p* value of less than 0.05 indicates significant pleiotropy in the genetic variants. Second, we applied MR‐PRESSO (Mendelian Randomization Pleiotropy RESidual Sum and Outlier) to identify and remove outlier SNPs that might introduce pleiotropic bias. Third, multivariable MR was performed adjusting for smoking exposure using pack‐years data from the IEU OpenGWAS database (Table S2) to account for this major confounder. Fourth, the Steiger directionality test was conducted to confirm that the causal direction runs from immune cells to cancer risk rather than reverse causation.

#### 2.5.2. Influence Analysis

Finally, the analysis assessed the impact of individual SNPs on the relationship between immune cells and oral and pharyngeal cancers by sequentially excluding single SNPs and recalculating the MR results for the remaining instruments. This leave‐one‐out stepwise exclusion process helps to identify and mitigate the influence of outliers or individual SNPs that might disproportionately affect the causal inference, thus enhancing the robustness and validity of the findings.

These comprehensive sensitivity analyses collectively ensure that our causal estimates are robust to various potential violations of MR assumptions, including weak instruments, horizontal pleiotropy, population stratification, and reverse causation. Results from all sensitivity analyses are reported in the Supporting Information.

### 2.6. Multivariate Mendelian Randomization (MVMR) Analysis and Mediating Effect Estimation

This research utilized MVMR as a way to expand on MR and evaluate how single and multiple exposures impact outcomes by examining genetic variants linked to various relevant exposures.

### 2.7. Cell Isolation and Preparation

The oral squamous cell carcinoma cell line CAL‐27 (National Infrastructure of Cell Line Resource, Beijing, China, Catalog #3131C0001000700122) was obtained from the National Infrastructure of Cell Line Resource (Beijing, China). This cell line was selected for its well‐characterized properties in oral cancer research, including authenticated morphological and molecular features, stable growth characteristics, and documented immunological responses. Cells were maintained in DMEM supplemented with 10% FBS and cultured at 37°C in a humidified atmosphere containing 5% CO_2_. PBMCs (LONZA, Basel, Switzerland, Catalog #CC‐2702) were purchased from LONZA (Basel, Switzerland) and processed according to the manufacturer′s instructions with strict adherence to temperature gradient control during thawing.

Two distinct immune cell populations were isolated from PBMCs using magnetic‐activated cell sorting (MACS) technology. For memory B cell isolation, a two‐step procedure was employed using the Memory B Cell Isolation Kit (Miltenyi Biotec, Cat# 130‐093‐546, Bergisch Gladbach, Germany). Naive CD8+ T cells were isolated using the Naive CD8+ T Cell Isolation Kit (Miltenyi Biotec, Cat# 130‐093‐244, Bergisch Gladbach, Germany). Both isolation procedures followed the manufacturer′s protocols. Cell purity was consistently >95% as verified by flow cytometry (antibody specifications detailed in Table S4).

### 2.8. Coculture Experiments

To investigate the interactions between immune cells and oral cancer cells, purified memory B cells and naive CD8+ T cells were cocultured with CAL27 cells at a ratio of 5:1 (immune cells to cancer cells) in 24‐well plates. The ratio was selected based on preliminary optimization experiments showing maximal dynamic range and on physiological relevance, as tumor‐infiltrating lymphocytes typically outnumber cancer cells in the tumor microenvironment. Additional validation was performed at 1:1 and 10:1 ratios to confirm key findings. The cocultures were maintained for 24, 48, and 72 h before analysis of cellular phenotypes, proliferation, and functional markers by flow cytometry.

### 2.9. Flow Cytometry Analysis

Cell proliferation, apoptosis, and phenotype were analyzed using a BD FACSCanto Flow Cytometer (BD Biosciences, San Jose, United States). For proliferation analysis, cells were labeled with 5 *μ*M CFSE (Invitrogen, Cat# C34554, Carlsbad, United States) and cultured for designated periods. Ki67 expression was assessed by intracellular staining using antihuman Ki67‐PE antibody (BD Biosciences, Cat# 556027, clone B56, San Jose, United States) following fixation and permeabilization. Apoptosis was evaluated using the Annexin V‐FITC/PI Apoptosis Detection Kit (BD Biosciences, Cat# 556547, San Jose, United States) according to the manufacturer′s instructions. Cells were resuspended in binding buffer and stained with 5 *μ*L Annexin V‐FITC and PI for 15 min at room temperature in darkness.

For phenotypic analysis, memory B cells were stained with antihuman CD69 (BioLegend, Cat# 310904, clone FN50, San Diego, United States) and antihuman CD80 (BioLegend, Cat# 305208, clone 2D10, San Diego, United States) antibodies. The purity of isolated cell populations was verified using antihuman CD19‐PE (BD Biosciences, Cat# 555413, clone HIB19, San Jose, United States), antihuman CD27‐APC (BD Biosciences, Cat# 558664, clone M‐T271, San Jose, United States), antihuman CD8‐FITC (BD Biosciences, Cat# 555366, clone RPA‐T8, San Jose, United States), and antihuman CD45RA‐PE (BD Biosciences, Cat# 555489, clone HI100, San Jose, United States) antibodies. For cytokine analysis, naive CD8+ T cells were analyzed for CD69 expression and intracellular cytokine production after PMA (Sigma‐Aldrich, Cat# P1585, St. Louis, United States) and ionomycin (Sigma‐Aldrich, Cat# I0634, St. Louis, United States) stimulation, using antihuman IFN‐*γ* (BioLegend, Cat# 502506, clone 4S.B3, San Diego, United States) and antihuman TNF‐*α* (BioLegend, Cat# 502908, clone MAb11, San Diego, United States) antibodies. Complete antibody specifications and dilutions are provided in Table S4.

### 2.10. Statistical Analysis

Data calculations and statistical analyses were conducted exclusively through R programming (https://www.r-project.org/, Version 4.3.2). Various statistical tests, including Cochran Q‐tests, leave‐one‐out analyses, and MR‐Egger intercepts, were employed to evaluate the strength and dependability of the findings and genetic pleiotropy. The analysis of MR and determination of causality direction was conducted using the TwoSampleMR software package (v0.5.7), including the Steiger directionality test. Additional analyses were performed using MR‐PRESSO (v1.0) and MendelianRandomization (v0.9.0) packages. The assessment criteria included OR and a 95% confidence interval (95% CI). Statistical analyses were conducted with a significant level of 0.05 on both sides. Multiple testing was corrected using FDR with Benjamini–Hochberg procedure, considering *q* − value < 0.1 as significant. All flow cytometry data were analyzed using FlowJo software v10.8.1 (BD Biosciences, San Jose, United States). Data from three independent experiments with five replicates each were presented as mean ± standard deviation.

## 3. Results

### 3.1. IV Screening

Our IV analysis identified 11 significant immune cell traits with robust genetic instruments. As shown in Table 1, all selected IVs demonstrated F‐statistics exceeding 10 (range: 30.09–2037.04), indicating strong genetic instruments. The highest median F‐statistic was observed for BAFF‐R on IgD+ CD38dim B cell (108.09), followed by CD127 on CD45RA+ CD4+ T cell (53.08), suggesting particularly reliable genetic instrumentation for these markers.

### 3.2. MR Causal Effect Estimation

**Figure 1:**
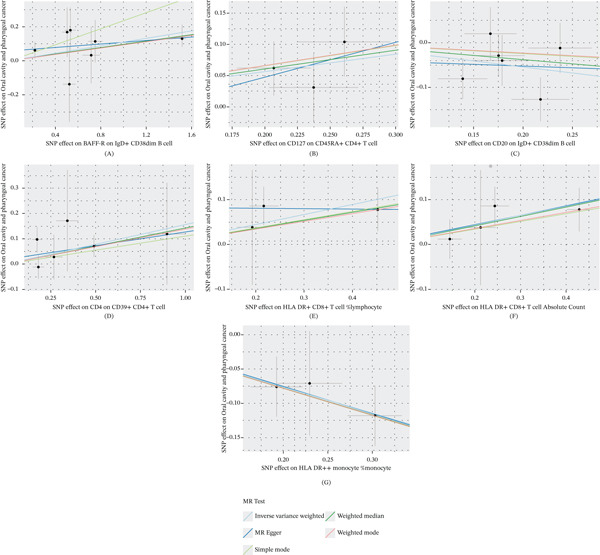
Effect estimates of different models of Mendelian randomization analysis of immune cells for oral and pharyngeal cancer. (A) BAFF‐R on IgD+ CD38dim B cell, (B) CD127 on CD45RA+ CD4+ T cell, (C) CD20 on IgD+ CD38dim B cell, (D) CD4 on CD39+ CD4+ T cell, (E) HLA DR+ CD8+ T cell %lymphocyte, (F) HLA DR+ CD8+ T cell absolute count, and (G) scatter plot of HLA DR++ monocyte %monocyte for different models of Mendelian randomization for oral and pharyngeal cancer.

The analysis employed five different models: MR Egger, WME, IVW, SM, and WM. Using IVW model significance (*p* < 0.05) as the screening criterion for significant causality, the causal effect estimations for all five models are presented in Table S3. The scatter plot of SNP effect estimation (Figure 1, showing results only when SNPs > 2) demonstrated that the fitting curves of all five models were similarly oriented, with consistent slopes across most models and the IVW model intercept approaching 0. The IVW analysis revealed several significant causal relationships between immune cell profiles and oral‐pharyngeal cancer risk (Table 1). The most notable associations included: Memory B cells showed the strongest risk association (OR = 3.44, 95% CI: 1.61–7.33, *p* = 0.001), indicating a substantial impact on cancer susceptibility. CD127 expression on CD45RA+ CD4+ T cells demonstrated a moderate risk effect (OR = 1.33, 95% CI: 1.04–1.70, *p* = 0.025), whereas HLA‐DR+ CD8+ T cells showed consistent associations across both percentages (OR = 1.25, 95% CI: 1.04–1.50) and absolute count measurements (OR = 1.24, 95% CI: 1.04–1.49). The Steiger directional analysis (Table 1) confirmed the validity of these causal relationships, with all identified associations showing correct directional effects (SNP *r* ^2^exposure > SNP*r* ^2^ outcome, *p* < 0.05). This directional consistency strengthens the reliability of our causal inferences.

### 3.3. Sensitivity Analysis

**Figure 2:**
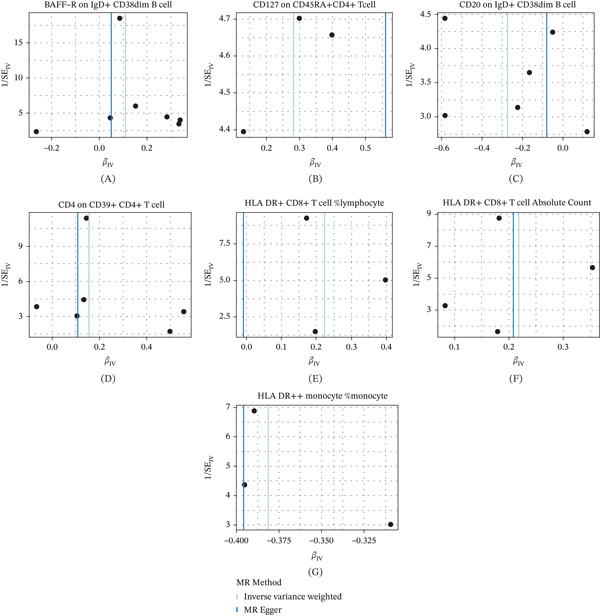
Funnel plot shows the heterogeneity test results for Mendelian randomization analysis of immune cells in relation to oral and pharyngeal cancer. (A) BAFF‐R on IgD+ CD38dim B cell, (B) CD127 on CD45RA+ CD4+ T cell, (C) CD20 on IgD+ CD38dim B cell, (D) CD4 on CD39+ CD4+ T cell, (E) HLA DR+ CD8+ T cell %lymphocyte, (F) HLA DR+ CD8+ T cell absolute count, (G) funnel plot of HLA DR++ monocyte %monocyte heterogeneity test for different models of Mendelian randomization for oral and pharyngeal cancer.

**Figure 3:**
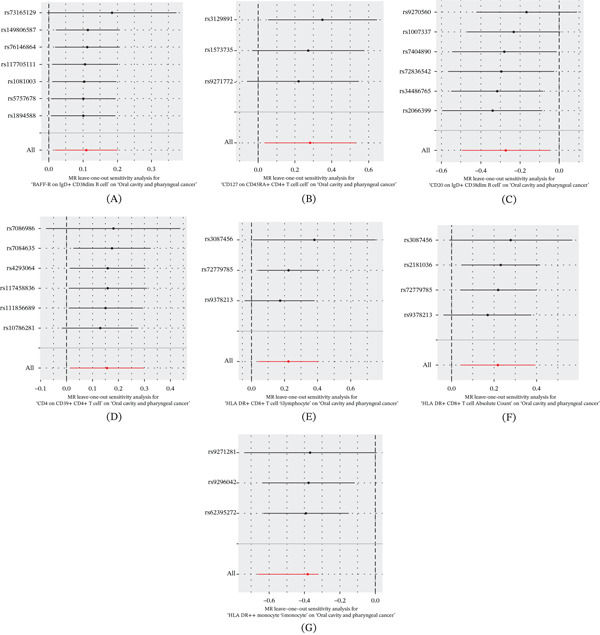
Mendelian randomization analysis of immune cells for oral and pharyngeal cancer one by one elimination test forest plot. (A) BAFF‐R on IgD+ CD38dim B cell, (B) CD127 on CD45RA+ CD4+ T cell, (C) CD20 on IgD+ CD38dim B cell in immune cells, (D) CD4 on CD39+ CD4+ T cell, (E) HLA DR+ CD8+ T cell %lymphocyte, (F) HLA DR+ CD8+ T cell absolute count, (G) HLA DR++ monocyte %monocyte forest plot of Mendelian randomization one‐for‐one elimination test for oral and pharyngeal cancer.

Heterogeneity assessment through Cochran’s Q test and *I* ^2^ statistics (Table 1) demonstrated robust consistency across genetic instruments. Most associations showed minimal heterogeneity, with *I* ^2^ values below 25% for key markers such as CD20 on IgD+ CD38dim B cells. The funnel plot analysis (Figure 2, showing results only when the number of SNPs > 2) demonstrated that the causal association effects were symmetrically distributed around the IVW model line, indicating no potential bias in the results. The MR‐Egger intercept analysis (Table 1) revealed no significant horizontal pleiotropy (all *p* values > 0.05), supporting the validity of our causal estimates. To further validate these findings, we conducted sensitivity analyses through a leave‐one‐out approach (Figure 3), where each line represents the effect size and 95% CI after removing individual SNPs, with the red line indicating the reference effect interval.

### 3.4. MVMR Analysis

MVMR analysis incorporating smoking‐related traits (Tables 2 and 3) revealed persistent immune cell associations after adjustment for smoking behaviors. Notably, CD127 on CD45RA+ CD4+ T cells (*β* = 0.261, *p* = 0.007), CD4 on CD39+ CD4+ T cells (*β* = 0.152, *p* = 0.009), and HLA‐DR+ CD8+ T cell markers maintained significant associations independent of smoking status, suggesting distinct causal pathways.

**Table 2:** Smoking‐related factors analysis and Steiger directionality test.

Smoking variable	Basic analysis					Steiger directionality test			
	SNPs	Beta	SE	OR (95% CI)	*p* value	SNP *r* ^2^ exposure	SNP *r* ^2^ outcome	Correct direction	Steiger *p* value
Maternal smoking around birth (ukb‐a‐39)	5	5.778	2.505	323.26 (2.38–43877.78)	0.021	0.000697	0.001518	FALSE	0.393865
Maternal smoking around birth (ukb‐b‐17685)	15	3.819	1.735	45.58 (1.52–1366.88)	0.028	0.001534	0.00417	FALSE	0.083472
Pack years of smoking	9	0.816	0.394	2.26 (1.04–4.90)	0.038	0.006238	0.002048	TRUE	0.0237
Smoking initiation	72	0.741	0.240	2.10 (1.31–3.36)	0.002	0.004796	0.020182	FALSE	5.32e‐07
Smoking status: never	59	1.506	0.764	0.22 (0.05–0.99)	0.049	0.006794	0.01079	FALSE	0.141871

Abbreviations: *β*, the effect coefficients in Mendelian randomization analysis; CI, confidence interval; OR, odds ratio; SNP, single nucleotide polymorphism.

**Table 3:** Results of multivariate Mendelian randomization analysis of the effect of smoking and immune cells on the incidence of oral and pharyngeal cancer.

Model	Exposure	Outcome	Beta	Standard error	*p*
Model 1	BAFF‐r on IgD +CD38dim B cell || id: ebi‐a‐GCST90001709	Oral cavity and pharyngeal cancer || id: ieu‐b‐90	0.087491	0.04605	0.057447
	Pack years of smoking PREVIEW ONLY || id: ukb‐a‐237	Oral cavity and pharyngeal cancer || id: ieu‐b‐90	0.887401	0.385726	0.021414
Model 2	CD127 on CD45RA +CD4 + T cell || id: ebi‐a‐GCST90001932	Oral cavity and pharyngeal cancer || id: ieu‐b‐90	0.260715	0.095968	0.006594
	Pack years of smoking PREVIEW ONLY || id: ukb‐a‐237	Oral cavity and pharyngeal cancer || id: ieu‐b‐90	0.812588	0.295537	0.005968
Model 3	CD20 + CD38dim on IgD B cell || id: ebi‐a‐GCST90001752	Oral cavity and pharyngeal cancer || id: ieu‐b‐90	0.11209	0.10701	0.294897
	Pack years of smoking PREVIEW ONLY || id: ukb‐a‐237	Oral cavity and pharyngeal cancer || id: ieu‐b‐90	0.927851	0.321062	0.003853
Model 4	CD4 on CD39 + CD4 +T cell || id: ebi‐a‐GCST90002061	Oral cavity and pharyngeal cancer || id: ieu‐b‐90	0.151986	0.057929	0.008699
	Pack years of smoking PREVIEW ONLY || id: ukb‐a‐237	Oral cavity and pharyngeal cancer || id: ieu‐b‐90	0.921348	0.307658	0.002747
Model 5	HLA DR on CD33 +HLA DR + CD14dim || id: ebi‐a‐GCST90002109	Oral cavity and pharyngeal cancer || id: ieu‐b‐90	0.23714	0.091631	0.009654
	Pack years of smoking PREVIEW ONLY || id: ukb‐a‐237	Oral cavity and pharyngeal cancer || id: ieu‐b‐90	0.805183	0.368588	0.028925
Model 6	HLA DR + CD8 + T lymphocyte cell % || id: ebi‐a‐GCST90001629	Oral cavity and pharyngeal cancer || id: ieu‐b‐90	0.214552	0.076055	0.004787
	Pack years of smoking PREVIEW ONLY || id: ukb‐a‐237	Oral cavity and pharyngeal cancer || id: ieu‐b‐90	0.883666	0.312774	0.004724
Model 7	HLA DR + CD8 + T cell Absolute Count || id: ebi‐a‐GCST90001627	Oral cavity and pharyngeal cancer || id: ieu‐b‐90	0.208506	0.069835	0.002829
	Pack years of smoking PREVIEW ONLY || id: ukb‐a‐237	Oral cavity and pharyngeal cancer || id: ieu‐b‐90	0.861134	0.299219	0.004003
Model 8	HLA DR++ monocyte % monocyte || id: ebi‐a‐GCST90001475	Oral cavity and pharyngeal cancer || id: ieu‐b‐90	0.35722	0.161644	0.02711
	Pack years of smoking PREVIEW ONLY || id: ukb‐a‐237	Oral cavity and pharyngeal cancer || id: ieu‐b‐90	0.612709	0.487568	0.208875
Model 9	IgD on IgD + CD38 + B cell || id: ebi‐a‐GCST90001824	Oral cavity and pharyngeal cancer || id: ieu‐b‐90	0.180396	0.122434	0.14064
	Pack years of smoking PREVIEW ONLY || id: ukb‐a‐237	Oral cavity and pharyngeal cancer || id: ieu‐b‐90	0.913893	0.354064	0.009847
Model 10	The Memory B cell % B cell || id: ebi‐a‐GCST90001406	Oral cavity and pharyngeal cancer || id: ieu‐b‐90	0.702769	0.383046	0.066552
	Pack years of smoking PREVIEW ONLY || id: ukb‐a‐237	Oral cavity and pharyngeal cancer | | id: ieu‐b‐90	0.724575	0.43775	0.097879
Model 11	Naive CD8 + T cell % T cell || id: ebi‐a‐GCST90001553	Oral cavity and pharyngeal cancer || id: ieu‐b‐90	0.01016	0.014354	0.479137
	Pack years of smoking PREVIEW ONLY || id: ukb‐a‐237	Oral cavity and pharyngeal cancer || id: ieu‐b‐90	0.913277	0.319359	0.00424

Abbreviations: *β*, the effect coefficients in Multivariate Mendelian randomization analysis; SNP, single nucleotide polymorphism.

### 3.5. Flow Cytometry Analysis

**Figure 4:**
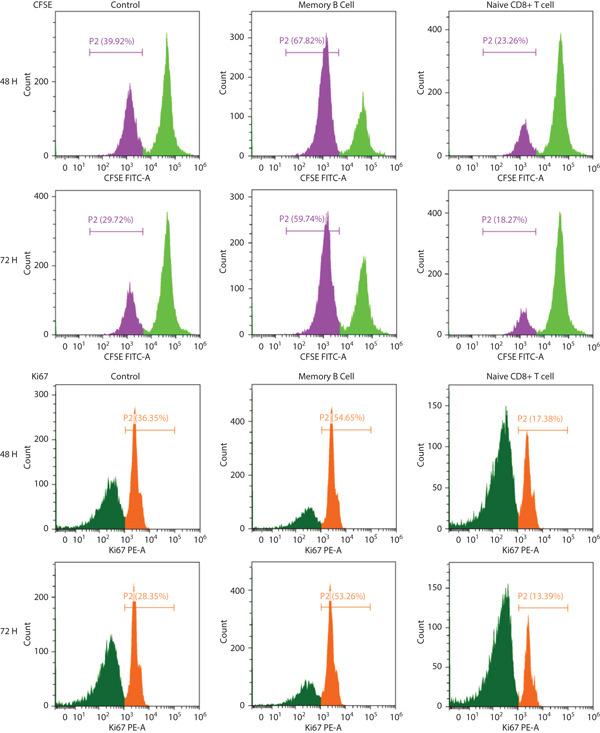
Effects of memory B cells and naive CD8+ T cells on oral cancer cell proliferation. Flow cytometry analysis showing Ki67 expression at 48 h and 72 h in three conditions: control, memory B cell coculture, and naive CD8+ T cell coculture. Memory B cell coculture showed significantly elevated Ki67‐positive rates (48 h: 54.65%, 72 h: 53.28%), whereas naive CD8+ T cell coculture exhibited substantially reduced rates (48 h: 17.38%, 72 h: 13.39%) compared with control (48 h: 36.35%, 72 h: 28.35%). Representative flow cytometry histograms demonstrate the differential effects of these immune cell populations on cancer cell proliferation.

Flow cytometry experiments provided crucial validation of our genetic findings. Cell proliferation analysis demonstrated that memory B cell coculture significantly enhanced cancer cell proliferation, with Ki67‐positive rates of 54.65% at 48 h and 53.26% at 72 h, compared with control rates of 36.35% and 28.35%, respectively. Conversely, naive CD8+ T cell coculture inhibited proliferation, reducing Ki67‐positive rates to 17.38% at 48 h and 13.39% at 72 h (Figure 4).

**Figure 5:**
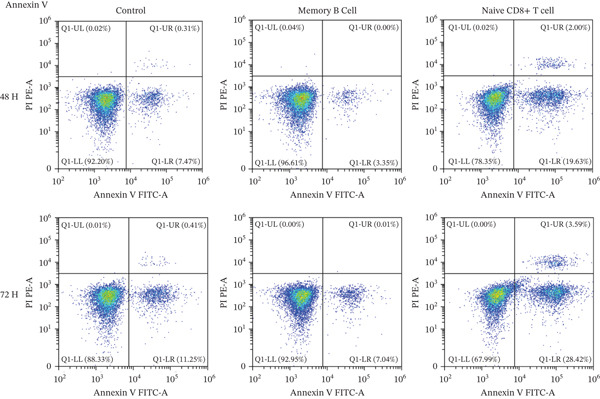
Analysis of cancer cell apoptosis in response to immune cell coculture. Annexin V/PI staining flow cytometry analysis at 48 h and 72 h demonstrates contrasting effects of memory B cells and naive CD8+ T cells on cancer cell apoptosis. Memory B cell coculture reduced apoptosis (viable cells at 72 h: 92.95%; early apoptosis: 7.04%), whereas naive CD8+ T cell coculture promoted apoptosis (viable cells at 72 h: 67.99%; early apoptosis: 28.42%) compared with control conditions. Quadrant analysis shows the distribution of viable, early apoptotic, and late apoptotic cell populations.

Apoptosis analysis through Annexin V/PI staining (Figure 5) revealed that memory B cells promoted cancer cell survival (92.95% viable cells at 72 h) while reducing apoptosis (7.04%). In contrast, naive CD8+ T cells induced significant apoptosis (28.42% at 72 h) and reduced cell viability (67.99%).

**Figure 6:**
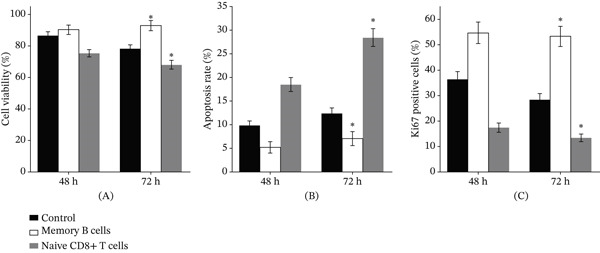
Flow cytometric characterization of immune cell populations associated with oropharyngeal cancer risk. Functional analysis of cell viability, apoptosis, and proliferation in immune cell subsets identified through Mendelian randomization. (A) Cell viability assessment demonstrating enhanced survival of memory B cells (white bars, 90.2*%* ± 3.0*%* at 48 h; 92.95*%* ± 3.2*%* at 72 h) compared with control PBMCs (black bars, 86.5*%* ± 2.5*%* at 48 h; 78.23*%* ± 2.1*%* at 72 h) and reduced viability of naive CD8+ T cells (gray bars, 75.3*%* ± 2.3*%* at 48 h; 67.99*%* ± 2.8*%* at 72 h). (B) Apoptosis rate analysis by Annexin V/PI staining showing decreased apoptotic cell death in memory B cells (5.2*%* ± 1.2*%* at 48 h; 7.04*%* ± 1.5*%* at 72 h) versus increased apoptosis in naive CD8+ T cells (18.5*%* ± 1.5*%* at 48 h; 28.42*%* ± 1.9*%* at 72 h) relative to control (9.8*%* ± 1.0*%* at 48 h; 12.35*%* ± 1.2*%* at 72 h). (C) Ki67 expression analysis revealing high proliferative capacity of memory B cells (54.65*%* ± 4.2*%* at 48 h; 53.26*%* ± 4.0*%* at 72 h) compared with suppressed proliferation in naive CD8+ T cells (17.38*%* ± 1.8*%* at 48 h; 13.39*%* ± 1.5*%* at 72 h) and intermediate levels in control cells (36.35*%* ± 3.1*%* at 48 h; 28.35*%* ± 2.5*%* at 72 h). Data represent mean ± standard deviation from *n* = 3 independent experiments with five replicates each. Statistical significance was determined by one‐way ANOVA with Tukey′s post hoc test. ∗*p* < 0.001 compared with control group.

Phenotypic analysis (Figure S2) showed that memory B cells exhibited progressive activation with increased CD69 (24 h 42.26% →72 h 71.63%) and CD80 expression, whereas naive CD8+ T cells demonstrated enhanced CD69 expression (24 h 51.85% →72 h 78.51%) and elevated cytokine production (IFN‐*γ*: 24 h 36.70% →72 h 67.77%; TNF‐*α*: 24 h 32.62% →72 h 45.97%). These experimental findings provide strong biological validation of the causal relationships identified through our genetic analyses, demonstrating the complex interplay between specific immune cell populations and cancer development. The visualization of flow cytometry data is shown in Figure 6.

## 4. Discussion

Our study provides compelling evidence for the complex interplay between specific immune cell populations and oral‐pharyngeal cancer risk through an innovative integration of genetic and experimental approaches. By leveraging MR analysis validated through detailed flow cytometry experiments, we demonstrated that memory B cells causally increase cancer risk while naive CD8+ T cells confer protection, with these associations persisting after smoking adjustment. These findings advance our understanding from correlative observations to causal relationships, revealing novel insights into how distinct immune cell subsets influence cancer susceptibility and progression [17, 18].

Although our study relied on surface marker expression rather than functional assays, these markers have established biological relevance in cancer immunology. CD127 expression correlates with T cell persistence and memory formation, CD39 identifies regulatory cells with suppressive function through adenosine generation, and HLA‐DR indicates activation status. Future studies incorporating functional assays such as cytotoxicity, cytokine production, and suppression assays would provide mechanistic validation of these phenotypic associations.

Most notably, our most striking finding was the strong association between memory B cells and increased cancer risk (OR = 3.44, 95% CI: 1.61–7.33), which was subsequently validated through coculture experiments showing enhanced cancer cell viability (92.95% at 72 h) and suppressed apoptosis. This observation aligns with recent studies demonstrating that tumor‐associated memory B cells can acquire immunosuppressive properties within the tumor microenvironment [21, 22]. Specifically, unlike previous assumptions about memory B cells′ protective role, our findings suggest their potential polarization toward a protumorigenic phenotype in oral‐pharyngeal cancer, possibly through interaction with Tregs and MDSCs [9]. Recent work further demonstrated that immune cell infiltration patterns in metastatic colorectal cancer correlate with patient survival outcomes, highlighting the broader relevance of immune cell dynamics in cancer progression [1].

Furthermore, the protective effect of naive CD8+ T cells (OR = 0.47, 95% CI: 0.30–0.75) was particularly noteworthy, with experimental validation showing significant induction of cancer cell apoptosis (28.42% at 72 h) and reduced viability (67.99%). This critical finding extends beyond previous correlative studies by establishing a causal relationship between Naive CD8+ T cell abundance and cancer risk reduction [11, 12]. Meanwhile, CD8+ T cell infiltration has been shown to correlate with distinct immune cell phenotypes based on comprehensive tumor immune profiling [3]. This aligns with findings from Bakkerus et al., who showed that higher CD8+ T cell infiltration in both peripheral blood and liver metastases was associated with improved survival in colorectal cancer patients [1]. Additionally, the observed high levels of IFN‐*γ* and TNF‐*α* production in our coculture experiments suggest that these cells maintain their capacity for antitumor responses even without prior antigen exposure [23], potentially through recognition of tumor‐associated molecular patterns.

In addition to these findings, the identification of CD127 expression on CD45RA+ CD4+ T cells as a risk factor (OR = 1.33, 95% CI: 1.04–1.70) provides a novel therapeutic target [24, 25]. Moreover, the persistence of this association after adjusting for smoking status (*β* = 0.261, *p* = 0.007) indicates a smoking‐independent mechanism of immune dysregulation.

Interestingly, HLA‐DR expression on CD8+ T cells showed consistent associations across percentage and absolute count measurements (OR≈1.25), suggesting a robust relationship with cancer risk [24, 25]. This marker may reflect T cell exhaustion within the tumor microenvironment, as supported by recent studies showing distinct exhaustion signatures in HLA‐DR+ CD8+ T cells from oral cancer patients.

From a clinical perspective, these findings have several immediate implications. First, monitoring memory B cell and naive CD8+ T cell ratios could serve as a novel prognostic tool, potentially identifying patients at higher risk of progression. Second, and perhaps more importantly, our results suggest that combination immunotherapy approaches targeting both B cell‐mediated immunosuppression and CD8+ T cell activation might be particularly effective [2, 7, 8]. This is supported by recent clinical evidence which demonstrated that immune cell profiles can serve as prognostic indicators even in advanced metastatic disease [1].

Of particular interest, the CD39+ CD4+ T cell association with cancer risk (OR = 1.17) merits special attention given recent discoveries about their role in adenosine‐mediated immunosuppression [26–28]. Consequently, our findings suggest that targeting the CD39/CD73 axis might be especially relevant in oral‐pharyngeal cancer, complementing emerging clinical data on adenosine A2A receptor antagonists.

Several important limitations merit discussion. First and most critically, our study is restricted to European‐ancestry populations. We fully acknowledge that this limitation significantly affects the generalizability of our findings, particularly given the pronounced geographic and ethnic variations in oral/oropharyngeal cancer incidence. Southeast Asia and East Africa exhibit substantially higher disease rates associated with region‐specific risk factors such as betel quid chewing, distinct tobacco usage patterns, and unique genetic susceptibilities. To address this limitation, we have initiated planning for follow‐up studies that will specifically target these high‐incidence regions. These future investigations will utilize region‐specific GWAS data as they become available and will incorporate local environmental and behavioral risk factors into the analytical framework. Although our study has provided valuable insights, we acknowledge certain limitations, particularly the focus on European populations in the genetic analysis, necessitating validation in diverse ethnic groups [18]. Second, we analyzed circulating immune cells which may not reflect the tumor microenvironment. Surface marker densities provide phenotypic but not functional characterization. The absence of HPV status, a major risk factor for oropharyngeal cancer, prevents stratified analysis by this critical variable. Third, despite robust individual SNP F‐statistics (all exceeding 30), the heterogeneity among genetic instruments is reflected in the difference between individual and pooled estimates. Although we performed extensive sensitivity analyses, residual confounding through unmeasured pathways remains possible. However, the robust experimental validation of key findings suggests the broad biological relevance of the identified mechanisms.

Recognizing the limitations of our European‐ancestry study, we are actively developing collaborative networks with research institutions in Southeast Asia and East Africa to conduct parallel investigations in these high‐risk populations. These studies will not only validate our current findings but also identify population‐specific immune markers that may be unique to these regions. By integrating genetic data with regional environmental factors and lifestyle variables, we aim to develop comprehensive risk prediction models that are applicable across diverse populations. This global approach is essential for achieving equitable advances in oral and pharyngeal cancer prevention. As we look to the future, several key research directions emerge from our findings. Single‐cell spatial transcriptomics could reveal the temporal dynamics of immune cell state transitions during cancer development, particularly focusing on the memory B cell polarization process we have identified. Furthermore, investigation of metabolic cross‐talk between identified immune cell populations and cancer cells might reveal new therapeutic vulnerabilities [10]. Additionally, prospective studies examining how these immune cell profiles evolve during immunotherapy could help optimize treatment selection and timing.

Our findings suggest several clinical applications. Immune profiling using CD127, CD39, and HLA‐DR expression could identify high‐risk individuals for enhanced screening. Therapeutic modulation of memory B cells or augmentation of naive CD8+ T cell responses represents potential preventive strategies. These markers could also guide patient selection for immunotherapy trials. Implementation would begin with prospective validation in high‐risk cohorts, followed by biomarker‐stratified prevention trials.

In conclusion, our integrated genetic and experimental approach has uncovered novel causal relationships between specific immune cell populations and oral‐pharyngeal cancer risk, with immediate implications for immunotherapy strategies. Most significantly, the identification of targetable mechanisms, particularly involving memory B cells and CD127+ CD4+ T cells, provides a strong foundation for developing more effective immunotherapeutic approaches in oral‐pharyngeal cancer [8–10]. Through continued research in these directions, we can work toward more personalized and effective immunotherapy strategies for oral‐pharyngeal cancer patients.

## 5. Conclusion

This study has made strides in enhancing our knowledge about how the immune system contributes to oral and pharyngeal cancer by making innovative use of MR. The discovery of links could open up new avenues for research and clinical intervention emphasizing the importance of considering the role played by the immune system in cancer pathogenesis. This research does not provide valuable insights into the intricate dynamics of cancer immunology but also lays a foundation, for future investigations that have tremendous potential to transform our approach to treating and preventing cancer.

## Author Contributions

X.D. contributed to conceptualization, design, data collection, statistical analysis, and drafted and critically revised the manuscript. S.H. contributed to project management and supervision.

## Funding

This study was supported by the Science Research Cultivation Program of Stomatological Hospital, Southern Medical University (PY2023010).

## Disclosure

All authors gave final approval and agreed to be responsible for all aspects of this study.

## Conflicts of Interest

The authors declare no conflicts of interest.

## Supporting Information

Additional supporting information can be found online in the Supporting Information section.

## Supporting information


**Supporting Information 1** Figure S1: Technology roadmap. This study presents schematic diagrams of multivariable Mendelian randomization and mediation analysis, adhering to the three criteria assumptions of association, independence, and exclusion restriction: (1) The selected instrumental variables are significantly associated with immune cells; (2) the instrumental factors do not have a significant connection to possible variables that could impact exposure or results; (3) the instrumental variables affect the incidence of oral and pharyngeal cancer solely through the pathway “Instrumental Variable → Exposure → Outcome.” These assumptions validate the use of instrumental variables in establishing the causal relationships in the study. The study′s analytical methodology is illustrated in a flowchart that encompasses SNP (Single et al.), IVW (inverse‐variance weighting), MR (Mendelian randomization), and GWAS (genome‐wide association studies). This flowchart delineates the process from identifying SNPs as genetic instruments to the MR analysis, highlighting the systematic approach employed in the study to infer causal relationships from genetic data.

**Supporting Information 2** Figure S2: Cell proliferation and activation marker analysis in immune cell cocultures CFSE proliferation assay results at 48 h and 72 h (left panels) showing differential proliferation rates among control, memory B cell, and naive CD8+ T cell conditions. Memory B cells demonstrated progressive increases in activation markers CD69 and CD80 over time (24–72 h). Naive CD8+ T cells showed enhanced CD69 expression and elevated production of cytokines IFN‐*γ* and TNF‐*α*, with significant increases from 24–72 h. The data illustrate the distinct phenotypic changes and functional responses of different immune cell populations during cancer cell coculture.

**Supporting Information 3** Table S1: List of immune cell‐related indicators in GWAS data.

**Supporting Information 4** Table S2: List of smoking‐related traits from MRC IEU OpenGWAS database.

**Supporting Information 5** Table S3: Causal effect estimation results of five models in Mendelian randomization analysis for immune cells and oral‐pharyngeal cancer.

**Supporting Information 6** Table S4: Complete antibody specifications and dilutions.

## Data Availability

The genetic association data used in this study are publicly available through the MRC IEU OpenGWAS platform (https://gwas.mrcieu.ac.uk/). The GWAS summary statistics for oral and pharyngeal cancer can be accessed using GWAS ID: IEU‐B‐90. Immune cell trait data are available through the identifiers listed in Table S1. The processed data supporting the findings of this study are included within the article and its Supporting Information. The raw experimental data generated during the flow cytometry analyses are stored in a secure institutional repository at the Stomatological Hospital, Southern Medical University, and are available from the corresponding author upon reasonable request. Requests should be directed to corresponding author.
