# Supplementary figures and images for: Immune Cell Profiles and Novel Insights Into Cancer Risk: A Focus on Oral and Pharyngeal Cancer
Source: Hum Mutat. 2026 Jul 9;2026:6585175. doi: 10.1155/humu/6585175 (PMC13347225; doi:10.1155/humu/6585175)

A

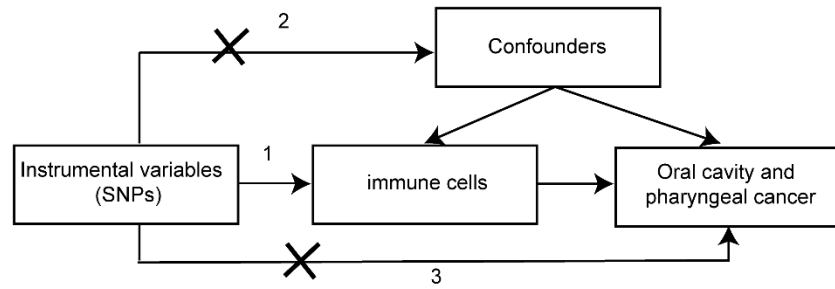

B

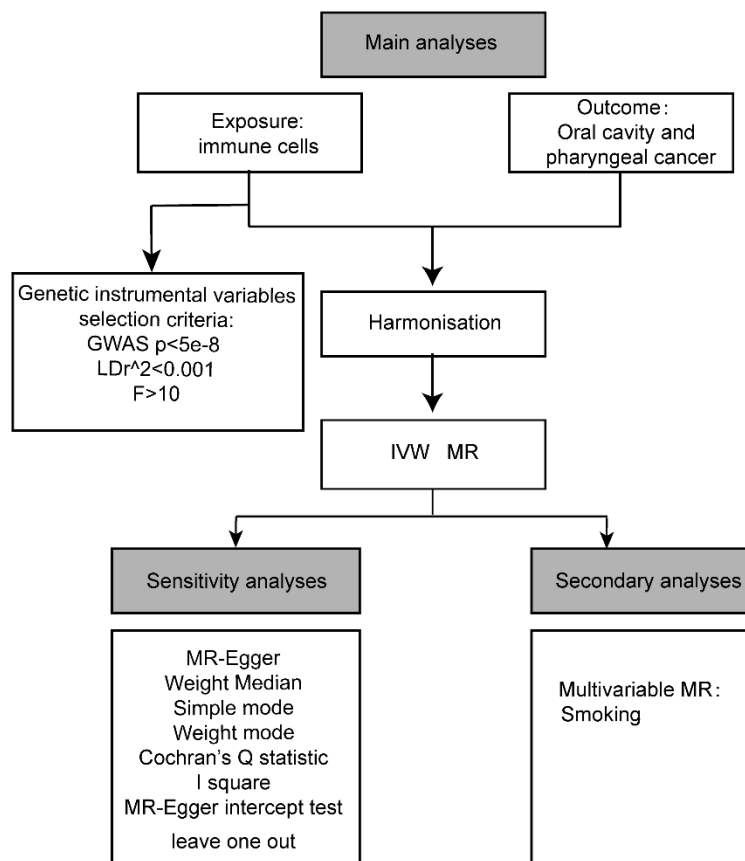

Supplement: Supplementary file 1 — Supporting Information 1 Figure S1: Technology roadmap. This study presents schematic diagrams of multivariable Mendelian randomization and mediation analysis, adhering to the three criteria assumptions of association, independence, and exclusion restriction: (1) The selected instrumental variables are significantly associated with immune cells; (2) the instrumental factors do not have a significant connection to possible variables that could impact exposure or results; (3) the instrumental variables affect the incidence of oral and pharyngeal cancer solely through the pathway “Instrumental Variable → Exposure → Outcome.” These assumptions validate the use of instrumental variables in establishing the causal relationships in the study. The study′s analytical methodology is illustrated in a flowchart that encompasses SNP (Single et al.), IVW (inverse‐variance weighting), MR (Mendelian randomization), and GWAS (genome‐wide association studies). This flowchart delineates the process from identifying SNPs as genetic instruments to the MR analysis, highlighting the systematic approach employed in the study to infer causal relationships from genetic data. [file HUMU-2026-6585175-s006.pdf]

# Memory B Cell

CD69

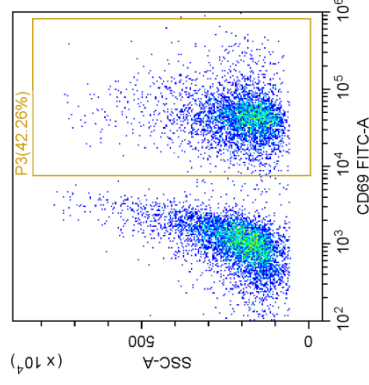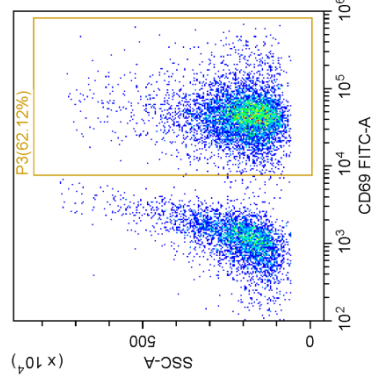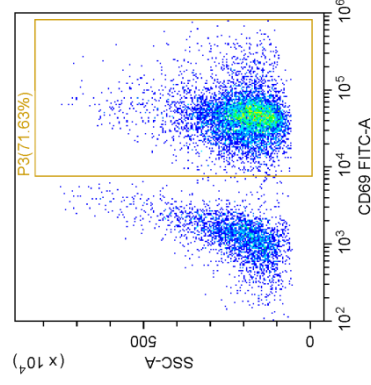

CD80

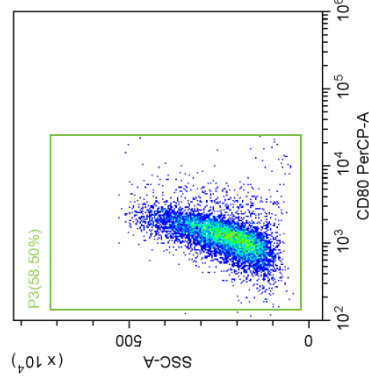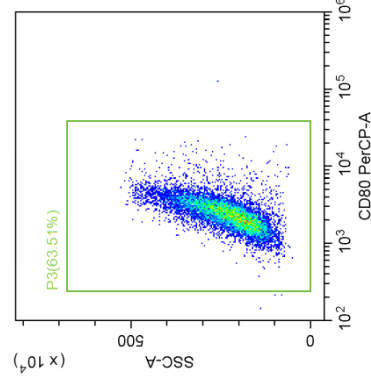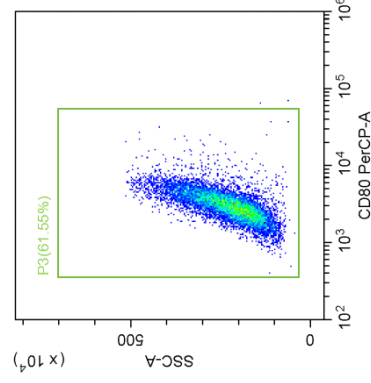

# Naive CD8+ T Cell

CD69

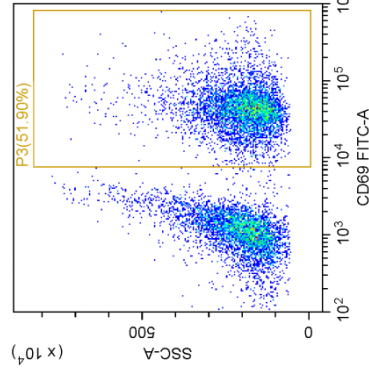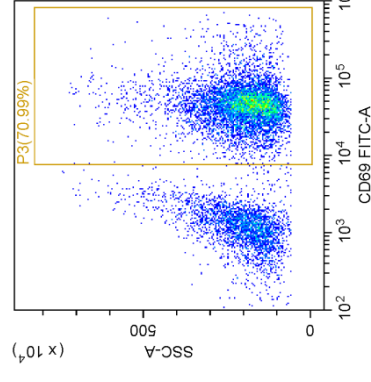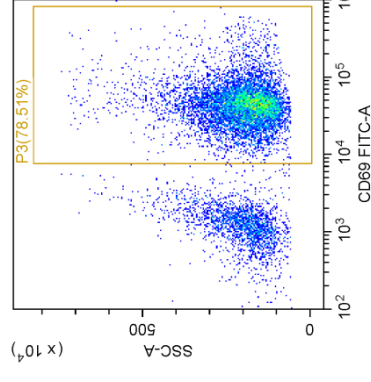

IFN- $\gamma$

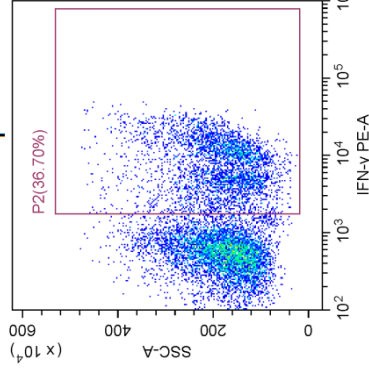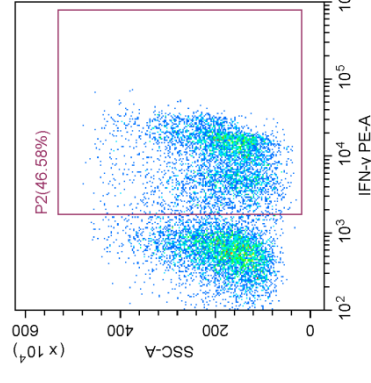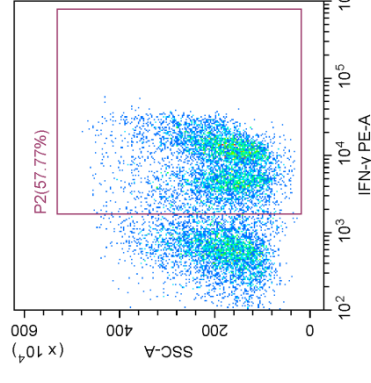

TNF- $\alpha$

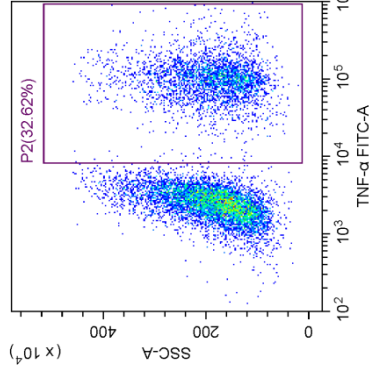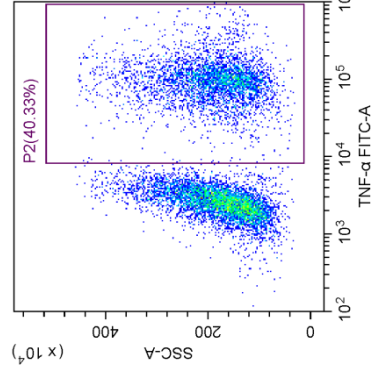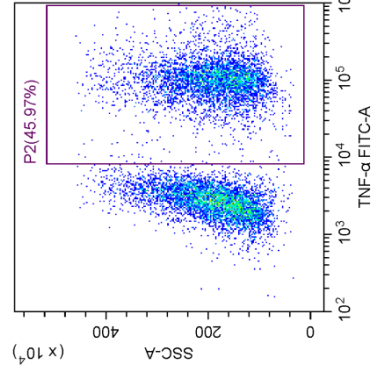

24H

48H

72H

Supplement: Supplementary file 2 — Supporting Information 2 Figure S2: Cell proliferation and activation marker analysis in immune cell cocultures CFSE proliferation assay results at 48 h and 72 h (left panels) showing differential proliferation rates among control, memory B cell, and naive CD8+ T cell conditions. Memory B cells demonstrated progressive increases in activation markers CD69 and CD80 over time (24–72 h). Naive CD8+ T cells showed enhanced CD69 expression and elevated production of cytokines IFN‐γ and TNF‐α, with significant increases from 24–72 h. The data illustrate the distinct phenotypic changes and functional responses of different immune cell populations during cancer cell coculture. [file HUMU-2026-6585175-s005.pdf]
